# Outcome Analysis Depending on the Different Types of Incision following Immediate Breast Reconstruction

**DOI:** 10.1155/2022/7339856

**Published:** 2022-02-01

**Authors:** Soo Hyun Woo, Jin Mi Choi, Jin Sup Eom, Eun Key Kim, Hyun Ho Han

**Affiliations:** Department of Plastic Surgery, Asan Medical Center, University of Ulsan College of Medicine, Seoul, Republic of Korea

## Abstract

**Background:**

Immediate breast reconstruction following nipple-sparing mastectomy (NSM) is widely used for its cosmetic benefits. Due to the lack of guidelines, the types of incisions in NSM vary and which method is superior remains a debate. In this study, we hypothesized that the periareolar incision has a higher risk of complications, such as nipple-areolar complex (NAC) necrosis, than other incisions.

**Methods:**

A retrospective chart review was conducted and divided into three groups: the periareolar, radial, and lateral incision groups. The reconstruction method and complications of NAC necrosis, wound dehiscence, seroma, hematoma, infection, and reconstruction failure were investigated.

**Results:**

A total of 103 patients (periareolar incision (33%, *n* = 34), radial incision (39.8%, *n* = 41), and lateral incision (27.2%, *n* = 28)) who underwent NSM and immediate breast reconstruction from 2018 to 2020 were included. The reconstruction methods were direct-to-implant, DIEP flap, LD flap, and PAP flap, and there was all of which had no statistically significant difference between the groups regarding the reconstruction method (*p*=0.257). In terms of complications, there was no significant difference in NAC necrosis (29.4%, 19.5%, and 21.4%, in the periareolar, radial, and lateral groups, respectively; *p*=0.578), wound dehiscence, seroma or hematoma, infection, and reconstruction failure.

**Conclusion:**

Breast reconstruction following NSM through periareolar incision does not increase the incidence of complications, including NAC necrosis. However, since only Asian patients with low BMI were included, if an appropriate patient group is selected for immediate reconstruction after NSM, reconstruction can be safely performed through the periareolar incision, and good cosmetic results can be obtained.

## 1. Introduction

There have been several changes in the field of total mastectomy for breast cancer. Unlike the conventional method of cutting off a large area of skin, the current technique preserves the skin as much as possible and demonstrates oncologic safety nonetheless [1–4]. In addition, with the increased prevalence of immediate breast reconstruction, attempts have been made to preserve the breast skin as much as possible. In particular, the nipple-sparing mastectomy (NSM), which also preserves the nipple-areolar complex (NAC), has recently become popular in clinical practice due to its aesthetic advantages [5, 6]. However, even if NSM has become a standard procedure for total mastectomy, various methods are used for the incision, depending on the breast surgeon because there are currently no guidelines on the incision method in NSM. The incisions made in patients with NSM include the periareolar incision, radial incision, lateral incision, and the inframammary fold (IMF) incision, each with its own advantages and disadvantages [7–9]. Here, complications, such as wound dehiscence and mastectomy skin flap necrosis, are common around the incision site. Therefore, depending on the location of the incision for mastectomy, the site of the complication varies, and this may affect the course of reconstruction.

For plastic surgeons performing breast reconstruction, the biggest concern is the necrosis of the NAC. Some plastic surgeons believe that performing mastectomy by making an incision around the areolar region or close to the NAC increases risks; there is much debate about this [5, 10]. In fact, when an implant or flap is placed under the mastectomy skin envelope, it becomes susceptible to necrosis as the NAC becomes the most apical site and, thus, receives the greatest pressure [11]. Considering these factors, the periareolar incision has a high risk of NAC necrosis. However, some breast surgeons still prefer it as it can hide scars [12].

A periareolar incision may lead to the necrosis of the NAC and a high probability of reconstruction failure. In this study, we compared the outcome with those of the incisions in other areas. Implant infection and explantation may increase in implant-based reconstructions, whereas flap handling through a small incision may potentially lead to flap failure in autologous-based reconstructions.

## 2. Materials and Methods

**Figure 1:**
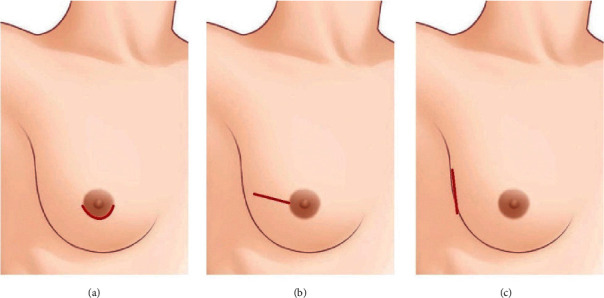
Incisions for nipple-sparing mastectomy. (a) Periareolar incision. An incision was made in the lower half along the border of the areolar. (b) Radial incision. (c) Lateral incision.

Patients who underwent NSM and immediate breast reconstruction at the Asan Medical Center between 2018 and 2020 were included in the study. Those who underwent the contralateral procedure and two-stage reconstruction with tissue expander/implant insertion were excluded. Of the 103 patients included in the study, 34 underwent NSM through the periareolar incision (incision was made in the lower half along the border of the areolar), 41 the radial incision, and 28 the lateral incision (Figure 1).

The data were collected after obtaining approval from the institutional review board, and a retrospective chart review was conducted. The patients' demographic information, specifically age, body mass index (BMI), comorbidities (i.e., hypertension and diabetes), smoking habits, neoadjuvant chemotherapy, postoperative chemotherapy, neoadjuvant radiotherapy (RT), postoperative RT, hormonal therapy, trastuzumab administration data, and grades of breast ptosis according to Regnault ptosis classification, was collected. The reconstruction weights (g) were recorded according to the type of NSM incision, reconstruction modality (direct-to-implant (DTI), deep inferior epigastric perforator (DIEP) flap, latissimus dorsi (LD) flap, or profunda artery perforator (PAP) flap), and mastectomy specimen weight (g). The postoperative complications were the primary outcome and included major necrosis of the NAC, minor necrosis, necrosis of the mastectomy skin, wound dehiscence, seroma, hematoma, and infections. Major necrosis referred to the cases in which surgical treatments, such as debridement and closure, were performed; minor necrosis referred to the cases in which secondary intention was treated without surgical treatment. The definition of reconstruction failure was the removal of the implant in the case of implant reconstruction or the removal of the flap in the case of flap reconstruction.

To compare the size of continuous variables (mean ± standard deviation), the two groups were analyzed using an independent *t*-test or Mann–Whitney *U* test. For categorical variables, the chi-square test or Fisher's exact test was used. The Kruskal–Wallis test was performed to confirm the significance between the three groups, and *p* < 0.05 was considered statistically significant. Statistical analysis was performed using SPSS 23.0 (IBM Corp., Armonk, NY, USA).

## 3. Results

The demographic data for the periareolar incision with (*n* = 3) or without (*n* = 31) extension group (Group 1, 33.0%, *n* = 34), radial incision group (Group 2, 39.8%, *n* = 41), and the lateral incision group (Group 3, 27.2%, *n* = 28) are presented in Table 1. The ages of those in Group 1 (39.26 ± 7.88 years) were significantly lower than those in Group 2 (49.80 ± 6.98 years, *p* < 0.001) and Group 3 (48.07 ± 8.24 years, *p* < 0.001). The number of patients who received neoadjuvant chemotherapy and postoperative chemotherapy was significantly higher in Group 2 than in Groups 3 and 1, respectively. No other significant difference was noted between Groups 1, 2, and 3, including ptosis grade.

Table 2 shows the reconstruction modality applied to each mastectomy incision group. The proportions of DTI, DIEP flap, LD flap, and PAP flap were 29.4% (*n* = 10), 50.0% (*n* = 17), 11.8% (*n* = 4), and 8.8% (*n* = 3) in Group 1 and 51.2% (*n* = 21), 41.5% (*n* = 17), 2.4% (*n* = 1), and 4.9% (*n* = 2) in Group 2, respectively. In Group 3, these were 57.1% (*n* = 16), 35.7% (*n* = 10), 3.6% (*n* = 1), and 3.6% (*n* = 1), respectively. There was no significant difference among the groups in the reconstruction modality according to the mastectomy incision (*p*=0.257).

The mastectomy specimen weight was significantly higher in Group 2 (360.55 ± 139.19 g) than in Group 1 (243.58 ± 123.21 g, *p* < 0.001) and Group 3 (272.00 ± 121.26 g, *p*=0.014). The reconstruction weights were not significantly different among the groups (*p*=0.426) (Table 3).

When complications were compared (Table 4), minor necrosis and major necrosis of the NAC occurred in 20.5% (*n* = 7) and 8.8% (*n* = 3) of the cases in Group 1; 17.0% (*n* = 7) and 2.4% (*n* = 1) of the cases in Group 2; and 10.7% (*n* = 3) and 10.7% (*n* = 3) of the cases in Group 3, respectively. Mastectomy skin necrosis developed in 2.9% (*n* = 1), 7.3% (*n* = 3), and 3.6% (*n* = 1) of the cases in Groups 1, 2, and 3, respectively. NAC necrosis showed a high percentage in Group 1, but not statistically significant (*p*=0.578). Even when NAC necrosis was divided into minor and major necrosis, there was no difference among the three groups (*p*=0.365; *p*=0.345). No significant difference among groups was noted in the incidence of mastectomy skin necrosis (*p*=0.635); wound dehiscence (3 cases (8.8%) in Group 1, two cases (4.9%) in Group 2, and two cases (7.1%) in Group 3 (*p*=0.793)); the case of seroma and hematoma (no cases (0%) in Group 1, one case (2.4%) in Group 2, and one case (3.6%) in Group 3 (*p*=0.572)); the incidence of surgical site infection (one case (2.9%) in Group 1, five cases (12.2%) in Group 2, and three cases (10.7%) in Group 3 (*p*=0.115)); and reconstruction failure, which occurred only once in the radial incision group (*p*=0.469).

## 4. Discussion

A topic of discussion when performing immediate breast reconstruction after NSM is whether preserving the NAC affects the oncologic safety and the possibility of NAC wound complications during reconstruction [13]. There are several pieces of evidence that support the oncologic safety of NSM. Wu et al. reported that the cancer recurrence rate in the NAC was low even after NSM was performed and that even if recurrence occurred in the NAC, there was no difference in the prognosis compared to the group of patients without recurrence if appropriate treatment was received [1]. They also found that there was no difference in the locoregional recurrence even if the tumor-to-nipple distance was less than 1 cm in the ductal carcinoma in situ patients who received NSM [3]. In particular, there are reports that have suggested that the locoregional recurrence in NAC is noninvasive compared to other sites [4]. In addition, if the NAC margin is pathologically negative, there is no reported difference in the results from the skin-sparing mastectomy [14, 15]. As there are pieces of evidence supporting such oncologic safety and the rate of early diagnosis of breast cancer has been increasing through the development and popularization of screening systems, the rate of NSM could only increase.

There are several different types of incisions used thus far in NSM. These include the periareolar incision, radial incision, lateral incision, IMF incision, mastopexy incision, reduction incision, and previous scar incision [7–9]. At our institution, breast surgeons mainly use the periareolar incision, radial incision, and lateral incision. In this study, these three incision types were compared because they are the most recently adopted incision types for NSM (Figure 1).

The controversy over which of the NSM incision types is superior still remains. According to Daar et al. [7], a meta-analysis of 8,729 NSM cases revealed that the incidence of partial NAC necrosis and full-thickness NAC necrosis was 3.14–6.37% and 1.87–3.21%, respectively. Among these, the periareolar incisions lead to a higher frequency compared to other types of incisions in terms of the reported incidence of necrosis in 6.82%, 8.25%, and 18.10% of the cases with IMF, radial, and periareolar incisions. Frey et al. analyzed the complications in patients who underwent NSM and reconstruction according to the type of incision and found that the rate of complications, such as mastectomy skin necrosis, was significantly higher in wise pattern and periareolar incisions [12]. Park et al. concluded that NAC necrosis occurred more frequently with periareolar incisions than with IMF and periareolar incisions [10]. Based on these results alone, the periareolar incision seems to yield a higher incidence of necrosis compared to other incisions.

However, there are studies with contrasting results. Paek et al. reported that there were no problems except for one case of hematoma in 34 patients who underwent NSM and reconstruction through the periareolar incision [16]. Peled et al. have reported that there was no significant difference in the NAC necrosis occurrence with the IMF, periareolar, and lateral incisions in patients who underwent postmastectomy RT [9]. Additionally, Park et al. reported that there was no significant difference in skin necrosis when comparing the periareolar and radial incisions [17]. Some reports found that the periareolar incision does not increase the rate of NAC necrosis; however, there are still many concerns regarding the stability of the incision around the NAC. Based on these findings from several studies, the IMF incision may seem to be the safest in terms of NAC necrosis; however, clinically, it is disadvantageous in that it is difficult to secure the upper pole view and to access the recipient vessel during autologous flap reconstruction [14]. In contrast, the periareolar incision is a method that is still used due to its cosmetic excellence as well as its enablement of the security of the field of view in mastectomy. It seems that the most preferred incision type has not yet been established, not only because of NAC necrosis but also because the type of immediate reconstruction method, the surgical field of view, and the cosmetic aspect must be considered in various ways.

**Figure 2:**
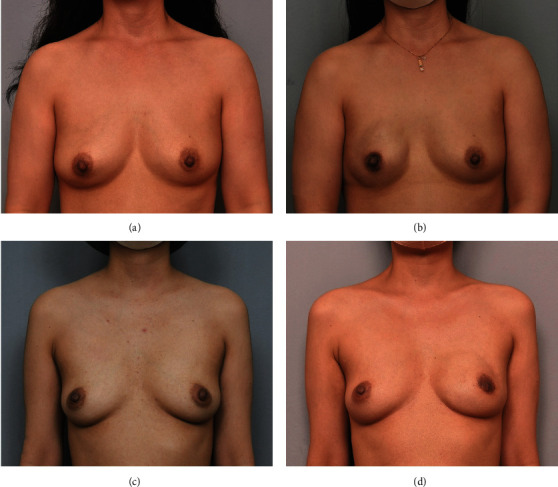
Pre- and postoperative clinical photos of direct-to-implant (DTI) reconstruction after periareolar incision. (a) Preoperative view. (b) Postoperative view at 1 year after DTI reconstruction of Rt. breast. There was no skin necrosis. (c) Preoperative view. (d) Postoperative view at 1 year after DTI reconstruction of Lt. breast. Areolar necrosis occurred, and debridement and closure were performed. Although the areola became small, the scar is not very visible.

**Figure 3:**
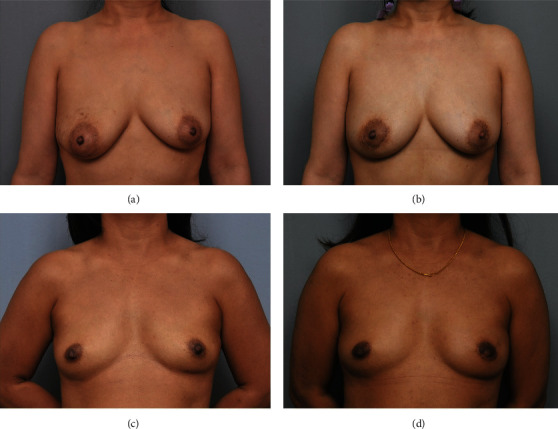
Pre- and postoperative clinical photos of deep inferior epigastric perforator (DIEP) flap reconstruction after periareolar incision. (a) Preoperative view. (b) Postoperative view at 1 year after DIEP flap reconstruction of Rt. breast. There was no skin necrosis. (c) Preoperative view. (d) Postoperative view at 1 year after DIEP flap reconstruction of Lt. breast with a small radial extension of the incision. The scar is well hidden.

Several concerns on periareolar incision are not simply because of NAC necrosis. In implant reconstruction, if NAC necrosis occurs, it is directly related to wound infection and leads to an increase in the probability of explantation. In autologous tissue reconstruction, since the incision is small, there is a difficulty in microscope work in a narrow field of view, and there is a concern about damage to the vessel in the process of inserting and withdrawing the flap through a relatively narrow space. However, there are still groups that opt to utilize the periareolar incision because the resulting scar is less noticeable, achieving cosmetically excellent results (Figures 2 and 3).

In this study, a retrospective chart analysis was performed under the assumption that the closer the mastectomy incision is to NAC, the more likely it is to affect NAC necrosis. In conclusion, there was no significant difference between NAC necrosis and mastectomy skin necrosis according to the type of mastectomy incision. In addition to skin necrosis, seroma, hematoma, infection, and wound dehiscence could also not be correlated with the incision type. In addition, in the case of breast ptosis, which is known as a risk factor for NAC necrosis through several studies [18, 19], there was no difference in ptosis grades between the three groups in this study. Therefore, in this study, the ptosis grade did not affect the selection of the incision type. The results can be interpreted as suggesting that among the currently used mastectomy incisions, there is no single superior method only in terms of complications. In particular, we found no significant difference in reconstruction failure in both implants and autologous tissue reconstruction with periareolar incision compared with other incisions. Therefore, it was inferred that the risks of complications and of reconstruction failure were not high compared to those with other incisions (Table 4).

This study had a few limitations. First was the population deviation. Since only a single Asian population was included in this study, it was a group with a relatively low BMI and a slim body habitus. Colwell et al. reported that smoking, high BMI, and preoperative RT can be seen as the predictors of skin necrosis in addition to the type of incision [20]. As such, BMI can be an important association factor for NAC necrosis, and, as such, a safe patient group was included in this study. However, the fact that there was no difference by incision site in the group of patients with a BMI of 30 or less among Asians suggests a clear clinical significance. Second, an even distribution of the cohort group was not obtained. Group 1 patients were relatively young, and the mastectomy weight of Group 2 was relatively high. This was because our breast surgeons, who performed the periareolar incisions, were doctors who mainly focused on young breast cancer patients (under 35 years of age), and young breast cancer patients tend to have a higher cancer stage at diagnosis; thus, the rate of postoperative chemotherapy is also high. Conversely, the rate of complications was not high for periareolar incisions despite the fact that the high cancer stage could also be a significant result.

Despite the limitations, we believe that factor control was relatively well-executed in this study compared to the clinical outcomes of other recent retrospective chart analyses as there was a large number of subjects (study population number) and as there were no intergroup differences in terms of the factors that affect mastectomy skin necrosis, such as BMI, smoking habits, and RT. The three types of mastectomy incisions and reconstruction modalities compared in this study are also the methods that are currently widely used, making the results of this study quite significant.

## 5. Conclusion

Immediate breast reconstruction through the periareolar incision after NSM does not increase the incidence of complications, including NAC necrosis. In particular, it does not affect the reconstruction failure. However, this applies to nonobese patients with a BMI of 30 or less; the risk of complications may be higher in obese patients. Therefore, reconstruction through the periareolar incision can be safely performed if an appropriate patient is selected, and excellent cosmetic results can be obtained as scars are not highly visible.

## Figures and Tables

**Table 1:** Patients' general demographic characteristics.

Variable	Group 1 (*n* = 34)	Group 2 (*n* = 41)	Group 3 (*n* = 28)	*p* value			
	Periareolar	Radial	Lateral	1 versus 2	2 versus 3	1 versus 3	1 versus 2 versus 3
Age (yr)	39.26 (7.88)	49.80 (6.98)	48.07 (8.24)	<0.001	0.451	<0.001	<0.001
Body mass index (kg/m^2^)	23.10 (3.73)	21.92 (1.89)	21.15 (1.62)	0.656	0.301	0.328	0.387
Height (m)	1.60 (0.05)	1.59 (0.05)	1.58 (0.06)	0.673	0.709	0.615	0.545
Weight (kg)	56.76 (7.39)	56.97 (6.11)	55.47 (6.75)	0.663	0.363	0.62	0.635
Hypertension	2 (5.9)	2 (5.0)	1 (3.6)	0.867	0.778	0.673	0.915
Diabetes mellitus	2 (5.9)	2 (4.9)	1 (3.6)	0.847	0.794	0.673	0.915
Current smoking	1 (2.9)	1 (2.4)	3 (10.7)	0.349	0.113	0.134	0.206
Neoadjuvant chemotherapy	12 (35.3)	23 (56.1)	9 (32.1)	0.072	0.05	0.794	0.08
Postoperative chemotherapy	3 (8.8)	11 (26.8)	4 (14.3)	0.046	0.215	0.499	0.108
Neoadjuvant radiotherapy	2 (5.9)	3 (7.3)	1 (3.6)	0.804	0.513	0.673	0.808
Postoperative radiotherapy	5 (15.2)	12 (30.0)	4 (14.3)	0.135	0.133	0.924	0.181
Hormone therapy	19 (55.9)	19 (46.3)	11 (39.3)	0.411	0.562	0.193	0.42
Trastuzumab therapy	5 (14.7)	4 (9.8)	3 (10.7)	0.511	0.897	0.641	0.789
Regnault ptosis classification							
Normal	21 (61.8)	23 (56.1)	17 (60.7)	0.646	0.806	1.000	0.908
Grade I	7 (20.6)	14 (34.1)	8 (28.6)	0.210	0.793	0.557	0.449
Grade II	6 (17.6)	4 (9.8)	3 (10.7)	0.497	1.000	0.494	0.392
Grade III	0 (0.0)	0 (0.0)	0 (0.0)	—	—	—	—

Values are presented as mean ± SD or number (%).

**Table 2:** Reconstruction modality.

Variable	Group 1 (*n* = 34)	Group 2 (*n* = 41)	Group 3 (*n* = 28)	*p* value
	Periareolar	Radial	Lateral	1 versus 2 versus 3
Implant	10 (29.4)	21 (51.2)	16 (57.1)	0.257
DIEP flap	17 (50.0)	17 (41.5)	10 (35.7)	
LD flap	4 (11.8)	1 (2.4)	1 (3.6)	
PAP flap	3 (8.8)	2 (4.9)	1 (3.6)	

Values are presented as number (%). DIEP, deep inferior epigastric perforator. LD, latissimus dorsi. PAP, profunda artery perforator.

**Table 3:** Mastectomy weight and reconstruction weight.

Variable	Group 1 (*n* = 34)	Group 2 (*n* = 41)	Group 3 (*n* = 28)	*p* value			
	Periareolar	Radial	Lateral	1 versus 2	2 versus 3	1 versus 3	1 versus 2 versus 3
Mastectomy weight (g)	243.58 (123.21)	360.55 (139.19)	272.00 (121.26)	<0.001	0.014	0.336	<0.001
Reconstruction weight (g)	266.40 (124.45)	327.00 (112.36)	260.00 (69.31)	0.286	0.366	1	0.426

Values are presented as mean ± SD.

**Table 4:** Complications.

Variable	Group 1 (*n* = 34)	Group 2 (*n* = 41)	Group 3 (*n* = 28)	*p* value			
	Periareolar	Radial	Lateral	1 versus 2	2 versus 3	1 versus 3	1 versus 2 versus 3
NAC necrosis	10 (29.4)	8 (19.5)	6 (21.4)	0.318	0.846	0.475	0.578
Minor necrosis	7 (20.5)	7 (17.0)	3 (10.7)	0.565	0.172	0.148	0.365
Major necrosis	3 (8.8)	1 (2.4)	3 (10.7)	0.221	0.149	0.802	0.345
Skin necrosis	1 (2.9)	3 (7.3)	1 (3.6)	0.401	0.513	0.889	0.635
Wound dehiscence	3 (8.8)	2 (4.9)	2 (7.1)	0.495	0.693	0.809	0.793
Seroma or hematoma	0 (0.0)	1 (2.4)	1 (3.6)	0.359	0.783	0.267	0.572
Surgical site infection	1 (2.9)	5 (12.2)	3 (10.7)	0.125	0.85	0.401	0.115
Reconstruction failure	0 (0.0)	1 (2.4)	0 (0.0)	0.359	0.413	1	0.469

Values are presented as number (%). NAC, nipple-areolar complex.

## Data Availability

The data that support the findings of this study are available from the corresponding author (HH Han).
